# Advances in the Pathogenesis of Diabetic Kidney Disease

**DOI:** 10.3390/ijms25084563

**Published:** 2024-04-22

**Authors:** Christodoula Kourtidou, Konstantinos Tziomalos

**Affiliations:** First Propedeutic Department of Internal Medicine, Medical School, Aristotle University of Thessaloniki, AHEPA Hospital, 54636 Thessaloniki, Greece

Diabetic kidney disease (DKD) is both a frequent complication of diabetes mellitus (DM) [1] and one of the main causes of chronic kidney disease (CKD) [2]. Currently, both the prevention and management of DKD focus on glycemic control and the treatment of other concurrent cardiovascular risk factors [1,3,4]. Beyond glycemic management, a more thorough comprehension of the molecular pathways underpinning DKD is important because it might result in the development of novel treatments [5]. The purpose of this Special Issue is to present the latest advances in the pathogenesis of DKD.

Long after glucose levels return to normal, there remains a perpetually elevated risk of diabetic complications, including nephropathy, due to metabolic memory of previous exposure to hyperglycemia [5,6]. This realization raises the possibility that factors other than genetic predisposition, particularly epigenetic mechanisms, participate in the pathogenesis of diabetic complications [5,6]. Epigenetic modifications are heritable traits that can affect a phenotype by interfering with gene expression without changing the DNA sequence [5,6]. Such epigenetic modifications include the cytosine methylation of DNA (DNA methylation), histone post-translational modifications (PTMs), and non-coding RNAs (microRNAs and long non-coding RNAs) [5,6]. The involvement of epigenetic regulation in DKD has been recently reviewed [7].

A systematic review demonstrated findings regarding microRNAs (miRNAs) in DKD [8]. This article reported that miR-21 and miR-29b were upregulated, while let-7a and miR-30e were downregulated, in patients with DKD [8]. In patients with DM, miR-377 was inversely associated with DKD, whereas miR-4536-3p was positively associated with DKD [8]. MicroRNAs let-7b-5p and miR-21-5p were positively correlated with progression to end-stage renal disease (ESRD), whereas let-7c-5p and miR-29a-3p were negatively associated with progression to ESRD in patients with DM [8]. The study concluded that miR-126 was inversely associated with the presence of DKD, microalbuminuria, and macroalbuminuria in patients with DM [8].

Ezzat et al. reported that miR-375 expression was decreased in participants with type 2 DM and macroalbuminuria in comparison with diabetic patients without nephropathy and diabetic patients with microalbuminuria [9]. The same study reported a positive correlation between the expression of miRNA-375 and serum transforming growth factor-β (TGF-β) levels in T2DM individuals, emphasizing its significance in the process of kidney fibrosis [9].

Dimuccio et al. reported that the levels of miR145 and miR126 were increased in urinary extracellular vesicles from patients with DKD in comparison with diabetic patients without nephropathy and that these further rose as albuminuria increased, noting their potential role in DKD progression [10]. In vitro, podocytes and renal tubular cells showed an elevation in both miR145 and miR126 levels in extracellular vesicles when subjected to epithelial to mesenchymal transition (EMT), suggesting an interaction between miRNAs and kidney fibrosis [10].

Hung et al. studied the effects of administration of GSK-J4, an inhibitor of methylation at histone H3 lysine 27 (H3K27), in streptozotocin (STZ)-induced diabetic mice [11]. The study demonstrated a reduction of proteinuria in STZ mice treated with GSK-J4 [11]. The investigators extended their research to assess possible correlations with kidney fibrosis [11]. In this regard, they found that the staining areas of collagen and fibrin accumulation in the glomerulus and the staining areas of fibronectin and collagen IV fibrosis-related proteins were reduced [11]. Moreover, the accumulation of α-SMA, fibronectin, and collagen IV proteins in the kidneys was reduced in STZ mice treated with GSK-J4 [11]. GSK-J4 administration also downregulated the mRNA expression of Dickkopf-1 (DKK1), TGF-β1, fibronectin, and collagen IV in the glomeruli of STZ mice [11].

Recent studies have proposed that epigenetic modifications are an essential component in the etiology of DKD. A number of studies have also linked epigenetic alternations with the pivotal phase of renal fibrosis. Further research is needed to establish the role of epigenetic modifications in the pathogenesis of DKD beyond their application as biomarkers. Investigation into epigenetic modifications is anticipated to provide novel insights about the pathogenesis of DKD and metabolic memory, which may then result in new diagnostic modalities, personalized treatments, and improved DKD outcomes.
